# HRVanalysis: A Free Software for Analyzing Cardiac Autonomic Activity

**DOI:** 10.3389/fphys.2016.00557

**Published:** 2016-11-22

**Authors:** Vincent Pichot, Frédéric Roche, Sébastien Celle, Jean-Claude Barthélémy, Florian Chouchou

**Affiliations:** ^1^EA SNA-EPIS 4607, Department of Clinical and Exercise Physiology, University of Lyon, Jean Monnet UniversitySaint-Etienne, France; ^2^NeuroPain Unit, Lyon Neuroscience Research Centre, Institut National de la Santé et de la Recherche Médicale U 1028/Centre National de la Recherche Scientifique UMR 5292, University of LyonLyon, France

**Keywords:** heart rate variability, autonomic nervous system, autonomic neuroscience, parasympathetic, sympathetic, RR interval variability, software

## Abstract

Since the pioneering studies of the 1960s, heart rate variability (HRV) has become an increasingly used non-invasive tool for examining cardiac autonomic functions and dysfunctions in various populations and conditions. Many calculation methods have been developed to address these issues, each with their strengths and weaknesses. Although, its interpretation may remain difficult, this technique provides, from a non-invasive approach, reliable physiological information that was previously inaccessible, in many fields including death and health prediction, training and overtraining, cardiac and respiratory rehabilitation, sleep-disordered breathing, large cohort follow-ups, children's autonomic status, anesthesia, or neurophysiological studies. In this context, we developed *HRVanalysis*, a software to analyse HRV, used and improved for over 20 years and, thus, designed to meet laboratory requirements. The main strength of *HRVanalysis* is its wide application scope. In addition to standard analysis over short and long periods of RR intervals, the software allows time-frequency analysis using wavelet transform as well as analysis of autonomic nervous system status on surrounding scored events and on preselected labeled areas. Moreover, the interface is designed for easy study of large cohorts, including batch mode signal processing to avoid running repetitive operations. Results are displayed as figures or saved in TXT files directly employable in statistical softwares. Recordings can arise from RR or EKG files of different types such as cardiofrequencemeters, holters EKG, polygraphs, and data acquisition systems. *HRVanalysis* can be downloaded freely from the Web page at: https://anslabtools.univ-st-etienne.fr *HRVanalysis* is meticulously maintained and developed for in-house laboratory use. In this article, after a brief description of the context, we present an overall view of HRV analysis and we describe the methodological approach of the different techniques provided by the software.

## Introduction

In the 18th century, Stephen Hales was the first to note that pulse fluctuations were related to blood pressure and respiratory rate (Lewis, 1994; Billman, 2011). However, not until the 1960s was interest renewed in spontaneous changes in cardiovascular parameters (Hon and Lee, 1963; Luczak and Laurig, 1973; Sayers, 1973; Billman, 2011). This opened the way to an understanding of cardiovascular regulation. Since then, considerable advances have been made in explorations of the autonomic nervous system (ANS) through examinations of heart rate variability (HRV)—or RR interval variability—which quantifies the successive variations in the interval from the peak of one QRS complex to the peak of the next, as shown on an electrocardiogram.

Although, the results interpretation has been the subject of intense criticism (Eckberg, 1997, 1998; Malik, 1998; Malliani et al., 1998; Sleight and Bernardi, 1998; Billman, 2013), the HRV method is widely used today (Rajendra Acharya et al., 2006; Chouchou and Desseilles, 2014). Generally speaking, cardiac activity is controlled by both the sympathetic and parasympathetic systems (Guyenet, 2006), which induce heart rate oscillations at different rhythms. The analysis of these oscillations allows assessing the initial efferent autonomic nervous activity. Pharmacological studies in animals and humans have identified physiological rhythm components of HRV (Sayers, 1973; Akselrod et al., 1981; Pomeranz et al., 1985). Overall, it has been shown that rapid changes of heart rate are due to parasympathetic activity, whereas slower changes are due to both parasympathetic and sympathetic activity. In this view, HRV analysis provides a non-invasive approach that allows examining physiological phenomena that were previously inaccessible, and which are often associated to health.

**Figure 1:**
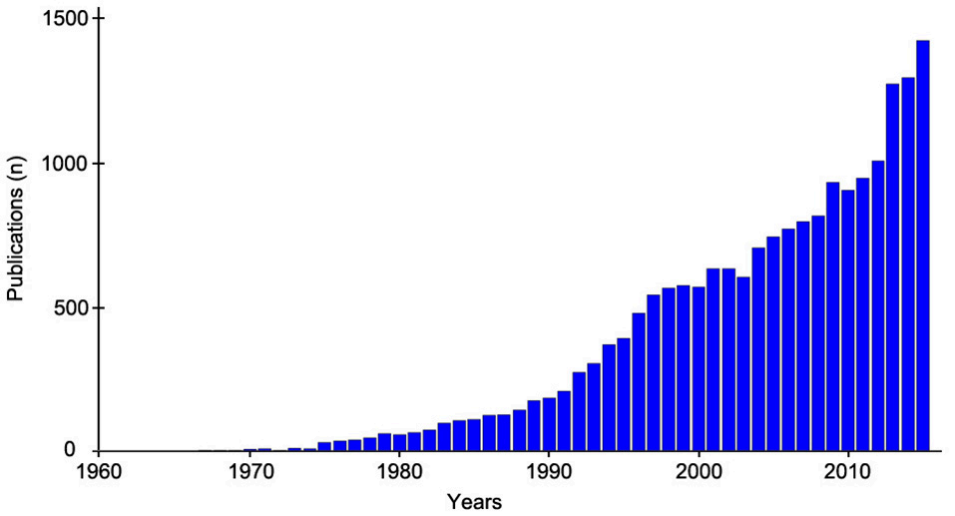
**Evolution of the annual number of publication related to Heart rate variability since 1960**. Medline search based on the terms “heart rate variability” or “RR intervals variablityi.”

Several methods for HRV quantification have been developed. They can be classified into frequency, temporal, nonlinear, and time-frequency analysis methods (Rajendra Acharya et al., 2006), and they are used in a variety of research fields, as evidenced by the growing number of publications on HRV analysis (Figure 1). Furthermore, HRV analysis indices have a wide range of applications, including research in physiology, cardiology, sport, health, mortality prediction, sleep, and pain, as well as for routine procedures in medicine and sports. The available data for HRV analysis therefore include RR interval series obtained from Holter systems or heart rate monitors or EKG data from acquisition or polygraph systems. Analyses can focus on short-duration recordings or 24-h recordings, and the data may concern small subject samples as well as large cohorts of hundreds or thousands of subjects, as in mortality prediction studies. Several HRV analysis methods are currently available, including Holter and heart rate monitor systems and free independent software such as Kubios (Tarvainen et al., 2014), Kardia (Perakakis et al., 2010), ARTiiFACT (Kaufmann et al., 2011), POLYAN (Maestri and Pinna, 1998), RHRV (Rodriguez-Linares et al., 2011), ECGLab (Carvalho et al., 2002), and the HRV Toolkit developed by the PhysioNet Project (Goldberger et al., 2000), each with its own specificity. Drawing on a large panel of users, we developed *HRVanalysis* (version 1.0), a software that includes a wide range of HRV analysis functions, from local to 24 h-recording, from single to large cohort files, and supporting various import formats. *HRVanalysis* is the outcome of almost 20 years of expertise, with each feature developed and improved for optimum adaptability to a variety of settings. The objective of this article is to provide an overall view of HRV analysis to a large community of users, to present the different proposed techniques along with their interests and limits, to detail the pre-processing of RR series, and, to present the wide possibilities of analysis allowed by the software.

## HRV: different methods for different types of exploration of cardiac autonomic function

### Time-domain analysis

Time-domain analysis is applied to quantify HRV using indices based on means or standard deviations, generally calculated over long-term recordings, typically 24 h (Kleiger et al., 1995; Balocchi et al., 2006; Rajendra Acharya et al., 2006). Because these indices represent short- to long-term variations in RR intervals, some indices are dependent on the recording length. It is therefore recommended to perform the calculations on recordings of similar duration to allow comparisons between measures or subjects.

The standard deviation of normal-to-normal intervals (SDNN) represents the variability over the entire recording period, giving the overall autonomic modulation regardless of sympathetic or parasympathetic arm. Some indices have been developed to quantify high frequency variations arising from parasympathetic activity such as the pNN50, calculated as the percent difference between adjacent normal RR intervals greater than 50 ms, and the rMSSD, calculated as the square root of the mean of the sum of the squared differences between adjacent normal RR intervals. The SDANN index, calculated as the standard deviation of the mean of all normal RR intervals for 5-min segments, quantifies the changes in heart rate due to cycles longer than 5 min. The SDNNIDX index, calculated as the mean of the standard deviation of all normal RR intervals for all 5-min segments, quantifies the changes in heart rate due to cycles shorter than 5 min (Task Force of the European Society of Cardiology and the North American Society of Pacing and Electrophysiology, 1996).

### Geometrical analysis

The geometrical indices are calculated on the sample density distribution of the RR intervals, which corresponds to the assignment of the number of equally long RR intervals to each value of their length (Malik, 1995; Task Force of the European Society of Cardiology and the North American Society of Pacing and Electrophysiology, 1996; Rajendra Acharya et al., 2006). The main studies that have validated this method have used bins of 7.8125 ms width that were adapted for RR series obtained from EKG at a sampling rate of 128 Hz. To allow comparisons, this bin width must be the same, regardless of the EKG sampling rate. Thus, for sampling rates other than 128 Hz, the sample density distribution graph presents discontinuities, and is then reconstructed by smoothing the curve using a moving average function. The HRV triangular index is calculated as the integral of the density distribution divided by the maximum of the density distribution (Y). The histogram can be interpolated as a triangle, using the minimum square difference. The triangular interpolation of the RR interval histogram (TINN) is the baseline width of this triangle.

These measures quantify overall HRV, and are influenced mainly by slow—but not rapid—oscillations of RR intervals (Malik et al., 1989). This geometrical method provides a good-quality analysis but requires a reasonable number of beats for effective application.

### Frequency-domain analysis: fourier transforms

The power spectral density (PSD) defined using Fourier transform is used to assess the different frequencies in the RR series. The spectrum is calculated using Welch's periodogram algorithm with a Hamming window of 256 points, an overlap of 50%, and at a precision of 256 points/Hz. The analyzed series can also be resampled from 1 to 8 Hz. The frequency indices are calculated as the integral of the PSD in specific bands of interest, as standardized by the Task Force in 1996 (Task Force of the European Society of Cardiology and the North American Society of Pacing and Electrophysiology, 1996). After that time, frequency-domain analyses of HRV were recommended to be performed on short-term 5-min recording segments and on long-term segments of typically 24 h. The two durations involve different bands of interest. The default bandwidths used to calculate the total power (Ptot), ultra-low frequencies (ULF), very low frequencies (VLF), low frequencies (LF), and high frequencies (HF) are summarized in Table 1. For short-term analyses, LF and HF are also expressed as normalized values LFnu = 100^*^LF/(Ptot-VLF) and HFnu = 100^*^HF/(Ptot-VLF), and the LF/HF ratio is calculated (Table 1).

**Table 1:** **Default bandwidth utilized for the calculation of frequency indices**.

**HRV analysis**	**Short-term**			**Long-term**		
**Age of subject**	**Newborn**	**Baby**	**Adult**	**Newborn**	**Baby**	**Adult**
Ptot (Hz)	0–2.00	0–1.40	0–0.40	0–2.00	0–1.40	0–0.40
ULF (Hz)	–	–	–	0–0.0001	0–0.003	0–0.003
VLF (Hz)	0–0.02	0–0.04	0–0.04	0.0001–0.02	0.003–0.04	0.003–0.04
LF (Hz)	0.02–0.20	0.04–0.15	0.04–0.15	0.02–0.20	0.04–0.15	0.04–0.15
HF (Hz)	0.20–2.00	0.15–1.40	0.15–0.40	0.20–2.00	0.15–1.40	0.15–0.40

Total spectral power (Ptot) indicates overall HRV, and is used to assess overall autonomic cardiac modulation as well as the SDNN parameter. HF power represents short-term HR variation. Studies have shown that injected atropine completely eliminated HF power (Akselrod et al., 1981; Pomeranz et al., 1985). Thus, HF power is modulated by parasympathetic activity only, corresponding to peak respiratory rate. Pharmacological studies have shown that muscarinic cholinergic blocker and beta-adrenergic blocker lowered LF power, enhanced by dual blockade (Akselrod et al., 1981; Pomeranz et al., 1985). Both parasympathetic and sympathetic cardiac activity would therefore be associated with HR power in the LF band. Saul et al. (1990) and others (Pagani et al., 1997) showed a concomitant increase in LF power and muscle sympathetic nerve activity measured by microneurography. Furthermore, under atropine, LF power increased during orthostatic testing (Taylor et al., 1998). Although, these studies showed sympathetic cardiac modulation in LF power, changes in LF power can be interpreted only in relation to HF power. Accordingly, normalized indexes such as the LF/HF ratio, LFnu, and HFnu are used to examine this relationship.

To summarize, whereas HF power is modulated by parasympathetic modulation, LF power is controlled by both sympathetic and parasympathetic activity, and normalized indexes or the LF/HF ratio are used to estimate sympathetic modulation and autonomic equilibrium (Pagani et al., 1984) although this last index remains criticized (Billman, 2013).

### Time-frequency transforms: transit changes in HRV

None of the above-described methods allow temporal localization of sudden changes in RR signal behavior. To overcome these limitations, we applied wavelet transform (Pichot et al., 1999), which obtains a temporally localized sliding analysis of the signal, providing access to the heart rate variability at any time, for example, when the autonomous nervous system equilibrium is suddenly disrupted by an acute clinical situation or a physiological laboratory intervention. This method has been used successfully to assess autonomic reactivity to tilt tests (Jasson et al., 1997), exercise (Tiinanen et al., 2009), sleep apneas (Chouchou et al., 2014), experimental pain (Chouchou et al., 2011), generalized interictal EEG discharge (Sforza et al., 2014), pharmacological blockades (Pichot et al., 1999) and, anesthesia (Pichot et al., 2001).

For the analysis, a temporal sliding window of different weights (corresponding to different analysis levels or frequency ranges) containing the wavelet function is shifted along the signal. The weight characterizes a family member with a particular dilatation factor. The calculation gives a serial list of wavelet coefficients, which represent changes in the correlation between the RR signal and a given wavelet at different levels of analysis (or different frequency ranges) along the signal. The correspondence between wavelet coefficients and frequencies allows calculating the standard, ULF, VLF, LF, HF, LFnu, HFnu, and the LF/HF ratio along time. A complete description of this method, which is implemented in the software, is available in two previously published articles (Pichot et al., 1999; Wiklund et al., 2002).

### A nonlinear approach: the complexity of HRV

An alternative, nonlinear approach was proposed to examine cardiac autonomic control (Voss et al., 1995). In recent years, interest has grown in the nonlinear dynamics that characterize autonomic cardiovascular control (Rajendra Acharya et al., 2006; Maestri et al., 2007b). Investigations of the complex feedback loops that impact the cardiac function have led to new indices designed to reflect signal complexity. We therefore incorporate certain of these indices that have proven useful for HRV interpretation and for health and mortality prediction (Maestri et al., 2007a; Huikuri et al., 2009).

#### Poincaré plot

The Poincaré plot, also called the Lorenz plot, is a graphic tool used to visualize a series of RR intervals (Kamen et al., 1996). SD1 and SD2 are calculated as the standard deviation of the distances of the RR intervals from the y = x line and the y = −x + 2^*^mean (RR) line, respectively. The SD1/SD2 ratio is also calculated. SD1 represents short-term RR variability and SD2 represents long-term variability.

#### Fractality

The analysis of the fractality of heart rate variability consist to quantify the repetition of patterns display at different scales. Many methods are available:

The detrended fluctuation analysis (DFA) is used to quantify the degree of self-similarity (fractuality) of the RR signal by calculating the average amount of RR fluctuation at different bin sizes. A detailed description of the method is provided in Peng et al. (1995). Briefly, the root-mean-square fluctuations of integrated and detrended RR series are measured across windows of different sizes and plotted according to the size of the observation window on a log-log scale. Two scaling exponents α_1_ and α_2_ are then calculated as the slope of the fluctuation = f (window size) line, for short- and long-term fluctuations, respectively.

Similarly, the Hurst exponent (H) measures the self-similarity of the RR signal. A fractal signal will result in an exponent value of around 1, a random signal will result in a value of 0.5, and strongly correlated signal behavior will result in a value of 1.5.

The power-law slope (ß) is influenced mainly by autonomic input to the heart. It quantifies the complexity of the RR interval in the long term, from minutes to hours. The ß index is the slope calculated on the PSD plotted on a log-log scale from 10^−4^ to 10^−2^ Hz (Bigger et al., 1996). The smaller the slope, the greater the loss of complexity. A value of −1.5 was determined to be the optimum threshold to predict mortality in the elderly (Huikuri et al., 1998; Makikallio et al., 2001).

Also, Higuchi and Katz algorithms were proposed to determine the fractal dimension of heart rate variability signal (Rajendra Acharya et al., 2006).

#### Entropy

Applied to heart rate variability, entropy is a measure of the regularity and complexity of pattern of different length. Entropy is high when the patterns are identicaly distributed and decreases if some patterns are more likely. Many indices have been proposed: the Shanon entropy and its derived indices (conditional entropy, corrected conditional entropy, normalized corrected conditional entropy) (Porta et al., 1998), the sample entropy (Richman and Moorman, 2000) and approximate entropy (Pincus and Goldberger, 1994). Another way to measure the rate of patterns recurrences in RR series is the Lempel-Ziv complexity (Ferrario et al., 2004).

#### Heart rate turbulence

Heart rate turbulence (HRT) denotes the fluctuations in RR following a single premature ventricular contraction (PVC) (Bauer et al., 2008), typically involving an early 2-to-3-beat acceleration phase, a late 10-to-20-beat deceleration phase, and a return to the base RR interval. The method consists of aligning and averaging all recorded PVCs in order to plot the PVC tachogram. The obtained pattern is characterized by two indices. Turbulence onset (TO) is the amount of acceleration following a PVC (i.e., the difference between the mean of the two RR intervals immediately following a PVC and the mean of the two RR intervals preceding the PVC). The turbulence slope (TS) corresponds to the steepest slope for five consecutive RR intervals within the 15 beats following a PVC. The TO quantifies the vagal withdrawal, and TS is explained by vagal reactivation (Bauer et al., 2008). HRT has been demonstrated a powerfull risk stratifiers after acute myocardial infarction (Schmidt et al., 1999). HRT calculation requires several PVCs, and is usually calculated on long-duration recordings, typically 24 h.

#### Deceleration and acceleration capacities

These two indices are used to estimate the vagal and sympathetic capacities by analyzing heart deceleration and acceleration capacity (Bauer et al., 2006). The method consists of finding all sequences of two successive RR beats that increase (deceleration) in order to align and average the segments surrounding the sequences. From the obtained pattern, the deceleration capacity (DC) is calculated as the difference between the mean of the two beats following deceleration and the mean of the two beats before deceleration. Similarly, the acceleration capacity (AC) is calculated by detecting all sequences that decrease. The DC index has been demonstrated to predict mortality after myocardial infarction (Bauer et al., 2006).

#### Largest lyapunov exponent

The largest Lyapunov exponent is used in nonlinear analysis of physiological signals for detecting chaos (Wolf et al., 1985). Indeed, its value will tend to zero for slowly varing RR signals, and will increases as the variations of the RR is higher (Acharya et al., 2004).

#### Symbolic dynamics

The principal of this analysis is to spread the chosen RR sequence on a fixed number of levels, to transform it into short patterns, to classified all patterns according to the direction of variation of the successive RRs (0V, 1V, 2LV, 2UV), and to evaluate their rates of occurrence (Porta et al., 2001). This method allows to study short heart rate variability pattern behavior. For example, the symbolic dynamics permitted to elucidate the neural pathophysiological mechanisms preceding acute cardiac events (Guzzetti et al., 2005).

#### Empirical mode decomposition

This principle of this analysis, adapted for nonlinear and non-stationar time series, is to decompose them into a limited number of oscillatory components (modes) from which are calculated the instantaneous frequencies (Balocchi et al., 2004). Then, the powers associated to selected modes corresponding to the low and high frequencies are calculated (pLF1, pLF2, pHF1, and pHF2), as well as the ratios between low and high frequency indices (IMAI1 and IMAI2).

### Limitations

Cardiac activity is controlled by the sympathetic and parasympathetic systems as well as hormonal system (Guyenet, 2006). These systems induce heart rate oscillations at different rhythms and HRV methods are used to study these rhythms and consequently autonomic cardiac modulations.

Slow and fast oscillations do not carry the same accuracy to represent sympathetic and parasympathetic activities. HRV analysis has been demonstrated reliable in assessing parasympathetic activity (Sayers, 1973; Akselrod et al., 1981; Pomeranz et al., 1985) while this method remains less accurate to assess sympathetic activity as heart rate slow changes are related partly to sympathetic and parasympathetic activity (Task Force of the European Society of Cardiology and the North American Society of Pacing and Electrophysiology, 1996). A better approach of sympathetic activity relies on examining relative changes in the LF/HF ratio and LF%, or SD1/SD2 (Pagani et al., 1984; Tulppo et al., 1996; Malliani et al., 1998). Several studies have shown that relative changes in fast- and slow-oscillations allow approaching relative sympathetic activity in response to tilt testing under atropine (Taylor et al., 1998) or experimental pain (Burton et al., 2009; Chouchou et al., 2011).

Furthermore, some indices, such as temporal indices for example, depend on the length of the selected RR series to analyse while other are not, such as PSD. Ideally, except for time-frequency analyses, the data should be stationary (Chouchou et al., 2009), not less than 5-min duration or 250 beats, recorded over comparable time period, and in similar situations as sitting, upright, lying for example (Bahjaoui-Bouhaddi et al., 2000).

Also, interpretation of HRV indices remains especially debated in long-term recordings, because major determinants of HRV such as environmental factors, physical activity, and sleep duration vary significantly over time (Lombardi and Stein, 2011). Thus, HRV indices can be calculated on the whole RR sample or as the mean of successive epochs. Users might be aware of that and can refer to Table 2 for these specifications.

**Table 2:** **HRV indices calculated in *HRVanalysis* program**.

				**Analysis type**			
**Index**	**Unit**	**Entire**	**Day/Night**	**Selected area**	**Along time**	**Arround events**	**Sequencial epoch**
**TEMPORAL**							
Mean RR	ms	x	x	x	x	x	x
Mean frequency	bpm	x	x	x	x	x	x
NN20	n	x	x	x			x
pNN20	%	x	x	x			x
NN30	n	x	x	x			x
pNN30	%	x	x	x			x
NN50	n	x	x	x			x
pNN50	%	x	x	x			x
SDNN	ms	x	x	x			x
rMSSD	ms	x	x	x			x
SDANN	ms	x	x				
SDNNIDX	ms	x	x				
**GEOMETRICAL**							
Triangular Index		x	x	x			x
TINN	ms	x	x	x			x
X	ms	x	x	x			x
Y	n	x	x	x			x
M	ms	x	x	x			x
N	ms	x	x	x			x
**FREQUENCY**							
Ptot	ms^2^/Hz	x	5 min	x			x
ULF	ms^2^/Hz	x					
VLF	ms^2^/Hz	x	5 min	x			x
LF	ms^2^/Hz	x	5 min	x			x
HF	ms^2^/Hz	x	5 min	x			x
LF/HF	–		5 min	x			x
LFnu	%		5 min	x			x
HFnu	%		5 min	x			x
**EMPIRICAL MODE DECOMPOSITION**							
pLF1		5 min	5 min	x			x
pLF2		5 min	5 min	x			x
pHF1		5 min	5 min	x			x
pHF2		5 min	5 min	x			x
IMAI1		5 min	5 min	x			x
IMAI2		5 min	5 min	x			x
**WAVELET**							
Ptot	ms^2^/Hz				x		
ULF	ms^2^/Hz						
VLF	ms^2^/Hz				x		
LF	ms^2^/Hz				x	x	
HF	ms^2^/Hz				x	x	
LF/HF	–				x	x	
LFnu	%				x	x	
HFnu	%				x	x	
**POINCARÉ PLOT**							
Centroïd	ms	x	x	x			x
SD1	ms	x	x	x			x
SD2	ms	x	x	x			x
SD1/SD2	–	x	x	x			x
SD1nu	%	x	x	x			x
SD2nu	%	x	x	x			x
**FRACTAL**							
α1_DFA_		5000 beats	5000 beats	x			x
α2_DFA_		5000 beats	5000 beats	x			x
H_DFA_		5000 beats	5000 beats	x			x
Hurst		5000 beats	5000 beats	x			x
H_Higuchi_		5000 beats	5000 beats	x			x
H_Katz_		5000 beats	5000 beats	x			x
1/f slope		x	x	x			x
**MOMENTS**							
Skewness		5000 beats	5000 beats	x			x
Kurtosis		5000 beats	5000 beats	x			x
Largest Lyapunov exponent				x			x
**ENTROPY**							
Approximate entropy		1000 beats	1000 beats	x			x
Sample entropy		1000 beats	1000 beats	x			x
Shanon Entropy (SE)		300 beats	300 beats	x			x
Conditional Entropy (CE)		300 beats	300 beats	x			x
Corrected CE (CCE)		300 beats	300 beats	x			x
Normalized CCE (NCCE)		300 beats	300 beats	x			x
ρ		300 beats	300 beats	x			x
Lempel-Ziv complexity		1000 beats	1000 beats	x			x
**TURBULENCE**							
VPC	n	x					
Turbulenve onset	%	x					
Turbulence slope	ms/nRR	x					
**DC/AC**							
Acceleration capacity	ms	x					
Deceleration capacity	ms	x					
**SYMBOLIC DYNAMIC**							
0V		300 beats	300 beats	x			x
0V%		300 beats	300 beats	x			x
1V		300 beats	300 beats	x			x
1V%		300 beats	300 beats	x			x
2LV		300 beats	300 beats	x			x
2LV%		300 beats	300 beats	x			x
2UV		300 beats	300 beats	x			x
2UV%		300 beats	300 beats	x			x
MP		300 beats	300 beats	x			x
MP%		300 beats	300 beats	x			x

When specified, the value of several indices is calculated as the mean of the successive epochs of indicated length (time duration or number of beats).

The software is optimized for analysing RR signal arizing from human recordings. R-peaks detection as well as HRV anayses can be performed on animal dataset by setting the adapted parameters in the preferences window. However, users must verify the accuracy of the methods for animals while some of them have been previously validated, such as Fourier analysis (Cerutti et al., 1991), others are not commonly use.

Finally, HRV analysis requires clean RR series while noisy recordings may lead to invalid physiological interpretations (Saul et al., 1988). An accurate preprocessing based on interpolation is usually utilize to correct for missing and extra beats.

## Signal processing and software description

**Figure 2:**
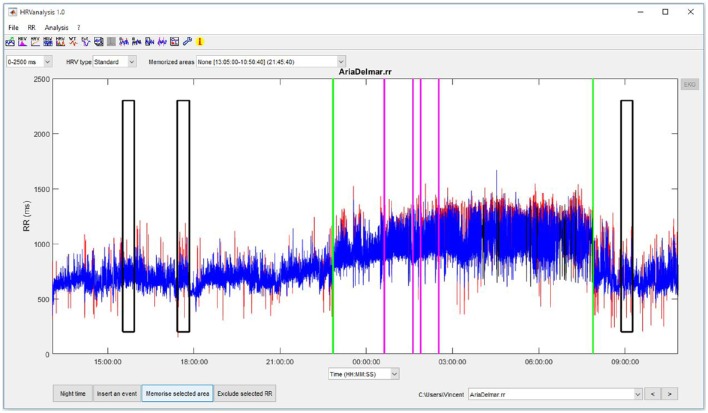
**Main figure of *HRVanalysis***.

The RR recording processing is decomposed in three data treatment steps: (1) RR importation or R peaks detection, (2) RR corrections and formatting, and (3) calculation of HRV indices (Figure 2). A tutorial containing a detailed description of the software is directly accessible from the main menu, and some sample runs for testing procedures are included with the software package.

### Data importation and R peaks detection

Data can be imported from EKG or RR files. The available formats for EKG data are EDF, ISHNE, binary, text, Matlab. A generic import window allows to pre-visualize the EKG trace while the user sets the data file specifications. Preformatted RR files can be imported from text and binary files. Several preformated configurations are proposed (Suunto, Polar, Aria Delmar, and Vista Novacor Holters).

R peaks are detected from EKG signals using a laboratory-developed algorithm largely inspired by Chen et al.'s method (Chen et al., 2006). This moving average-based method, initially developed for real-time QRS detection, has demonstrated a 99.5% detection rate in the MIT-BIH Arrhythmia Database. First, the raw EKG signals are upsampled at 1000 Hz to ensure good time resolution for the RR intervals, even at a low initial EKG sampling rate. The raw EKG signal is first denoised using wavelet transform. The denoised signal is then passed through a moving averaging-based linear high-pass filter in order to highlight the QRS complex, which is processed by full-wave rectification and nonlinear amplification followed by sliding-window summation. The resulting pulse train (i.e., waveform) is used to calculate an adaptive threshold for QRS complex detection.

### RR corrections and formatting

EKG recordings can contain ectopic beats—due to cardiac dysrhythmia—and missing or spurious beats—generally due to poor EKG signal quality. These nonsinusal beats must be corrected, because they induce errors in the calculation of HRV indices (Saul et al., 1988), which could result in invalid physiological interpretations.

In order to replace invalid beats, the software therefore provides automatic correction of the RR series, inspired by the algorithm developed by Kamath and Fallen (Kamath and Fallen, 1995). First, false beats are detected using Cheung's algorithm (Cheung, 1981): a high and low threshold are set for the relative variation in successive RR intervals (+32.5% and −24.5%, respectively). Second, for each detected error, the number of missing beats is estimated by comparing the total time duration within the error period with the duration of the immediately preceding beat. If the number of beats to recalculate is 3 or less, a cubic spline interpolation is done. This configuration generally originates from a single supraventricular or ventricular ectopic beat, or from an isolated R-peak missdetection. For 4 or more successive errors, the missing beats are interpolated by copying and inserting the same number of previous RRs between the first and last valid RR Although, these corrections generally results in a clean RR signal, this type of automatic algorithm can produce inconsistent values when the original EKG or RR signal is too corrupted, with lengthy portions of successive false beats. It is therefore recommended to visually inspect and review each series before performing HRV analyses. Additionally to RR correction, the software enables excluding parts of the signal from analysis, and the number of corrections is displayed in the results.

RR can also be manually corrected by two methods. First, the user can select an RR interval and change its value either by entering a new value or by using spline cubic interpolation to determine it. Second, when the EKG trace is available, R peaks can be manually inserted and removed by editing the EKG signal.

EKG recordings commonly contain excessive artifacted data that cannot be correctly interpolated. If the number of these data is low, HRV analysis can be performed on the remaining valid signals. *HRVanalysis* 1.0 allows excluding certain data from the analyses to avoid abnormal HRV values. This can be done by manually selecting the area to exclude or by using an automatic method based on successive beat-to-beat variations and RR labels (if present).

### Calculation of HRV indices

Calculations are performed according to the parameters set in the Preferences menu. For each type of analysis, a menu allows saving the results as a.txt file, saving the figure, and printing the figure. When saving the results, if the user selects an already existing.txt file, the data will be appended to the file. This allows combining analyses derived from different parts of the signal and/or different files and using the results directly in a statistical analysis program.

#### Linear and nonlinear HRV analysis of 24-h, day, and night periods

**Figure 3:**
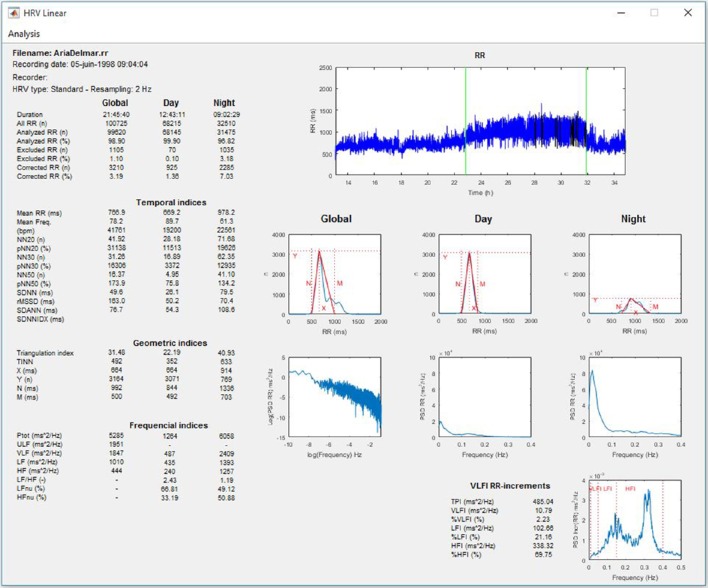
**Linear HRV analysis whole recording/day/night periods**.

**Figure 4:**
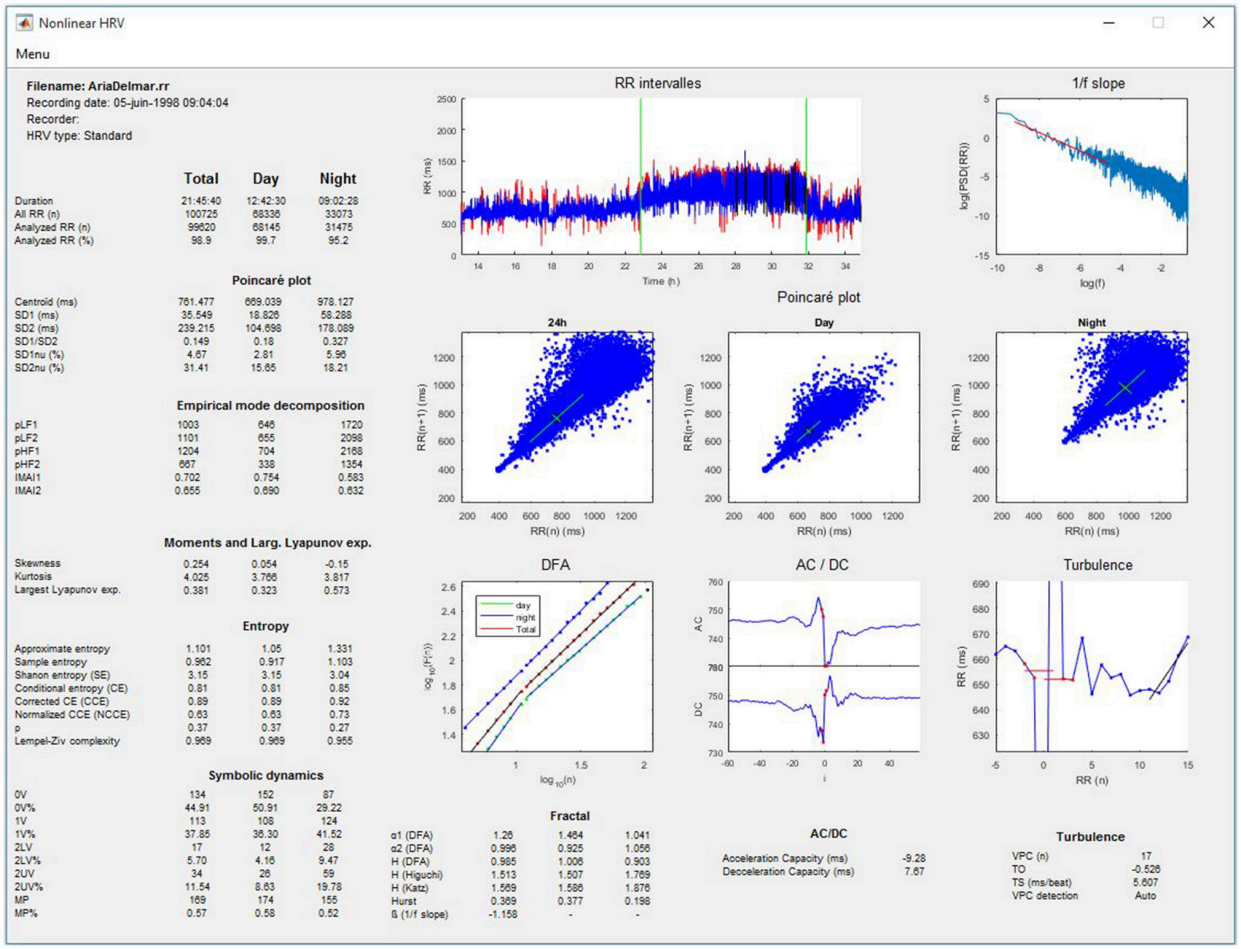
**Nonlinear HRV analysis whole recording/day/night periods**.

The software allows linear and nonlinear HRV analyses performed for long-term recordings, for example, using the 24-h ambulatory Holter system, as recommended by the Task Force (Task Force of the European Society of Cardiology and the North American Society of Pacing and Electrophysiology, 1996). In practice, the software allows analyses for RR series of durations longer than 2 h. If the night time has been entered, the analyses are computed for day, night, and entire recording periods (Figures 3, 4).

#### Sequential analysis of HRV

**Figure 5:**
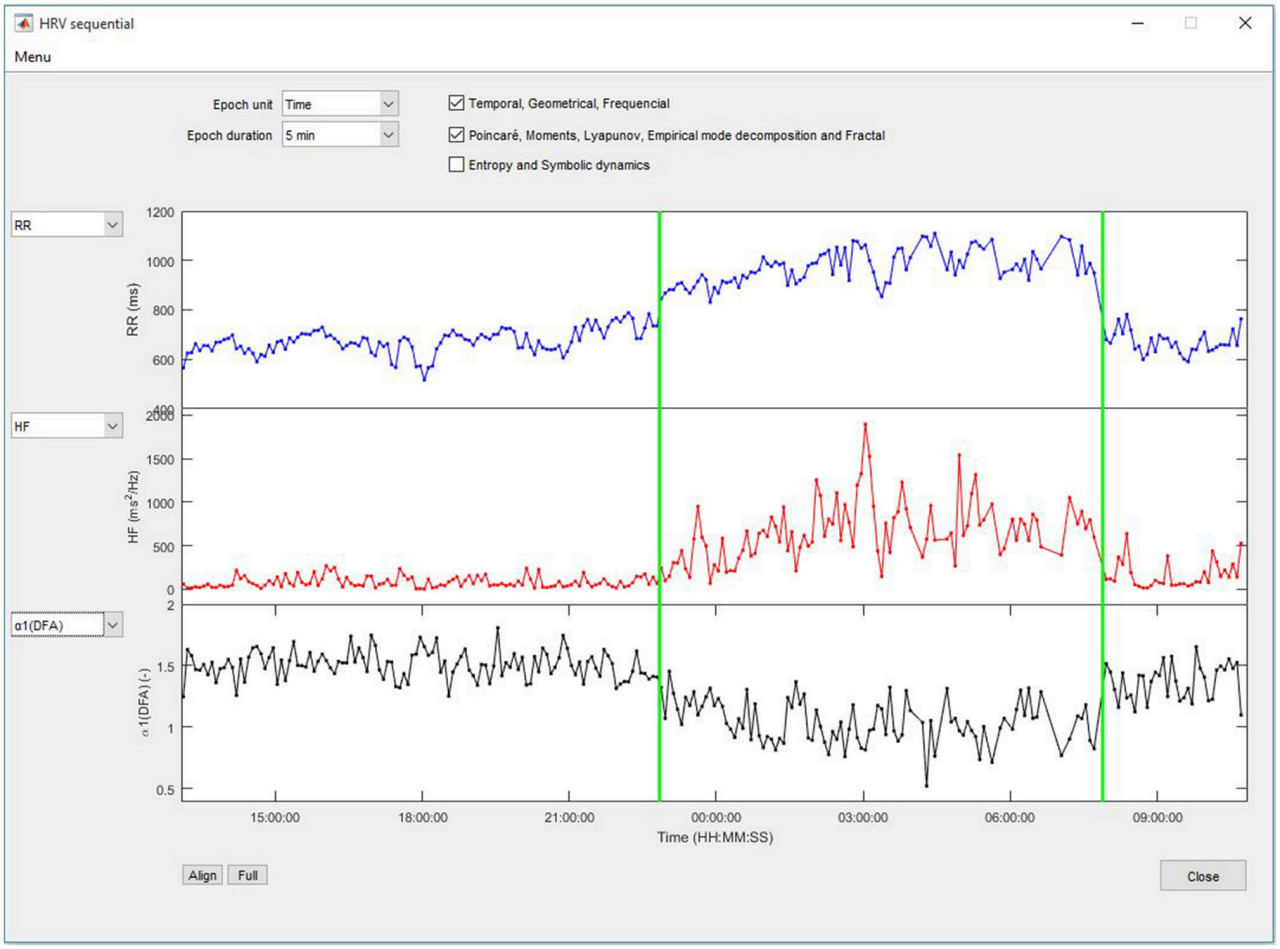
**HRV indices calculated on successive epochs**.

This analysis allows calculating changes in linear and nonlinear indices for successive epochs along the entire signal (Figure 5). The epoch duration is selected from a list of preset values ranging from 5 min to 1-h duration or from 250 to 4096 beats. If an epoch contains excluded RR intervals, the HRV calculation is not performed on the portion of the signal.

#### Local analysis

##### HRV indices

**Figure 6:**
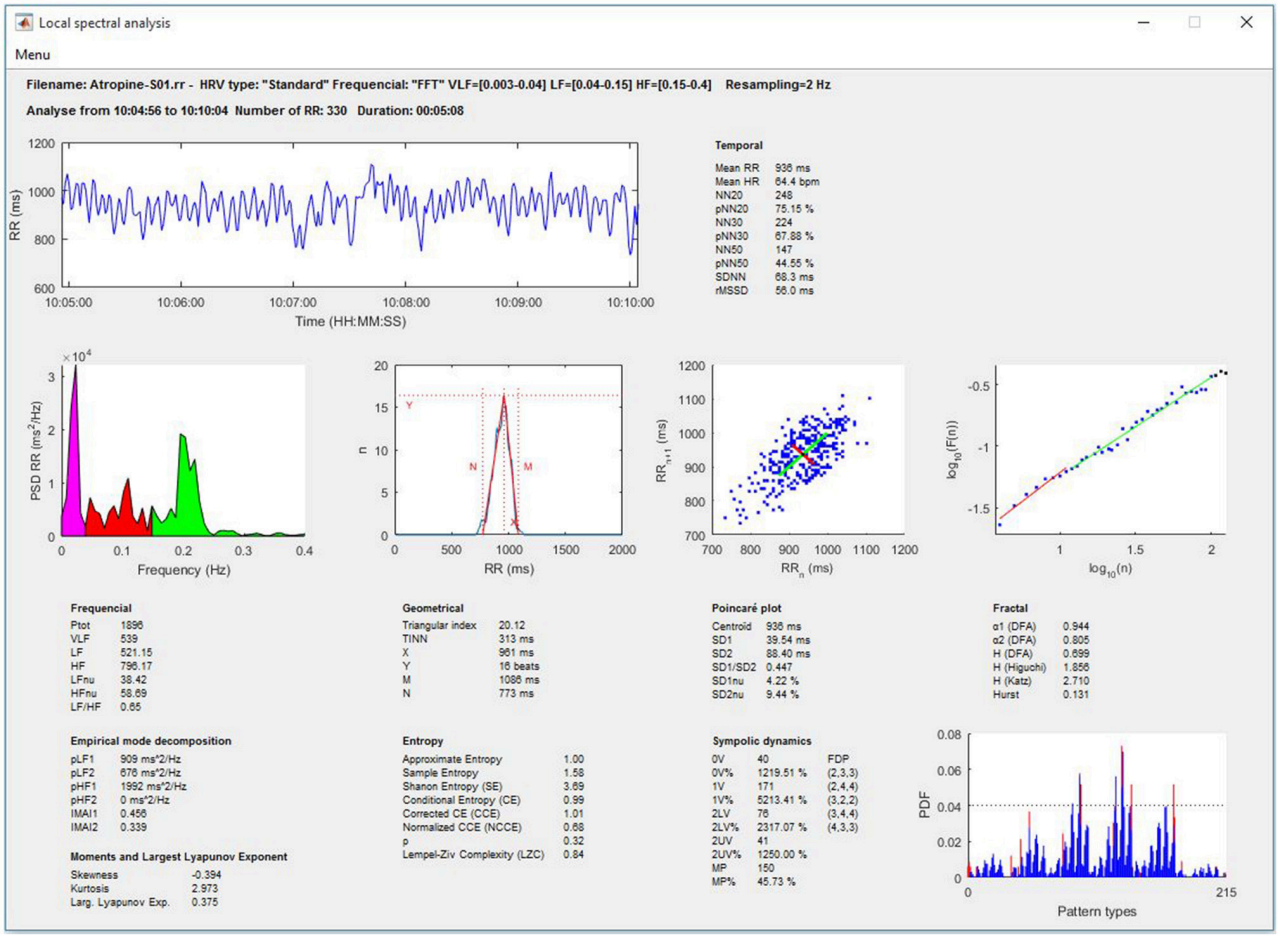
**Local linear and nonlinear analysis on a selected portion of the recording**.

This analysis calculates the linear and nonlinear indices of HRV for the RR part of the signal which is selected from the total RR signal (Figure 6).

##### Time-frequency analysis

**Figure 7:**
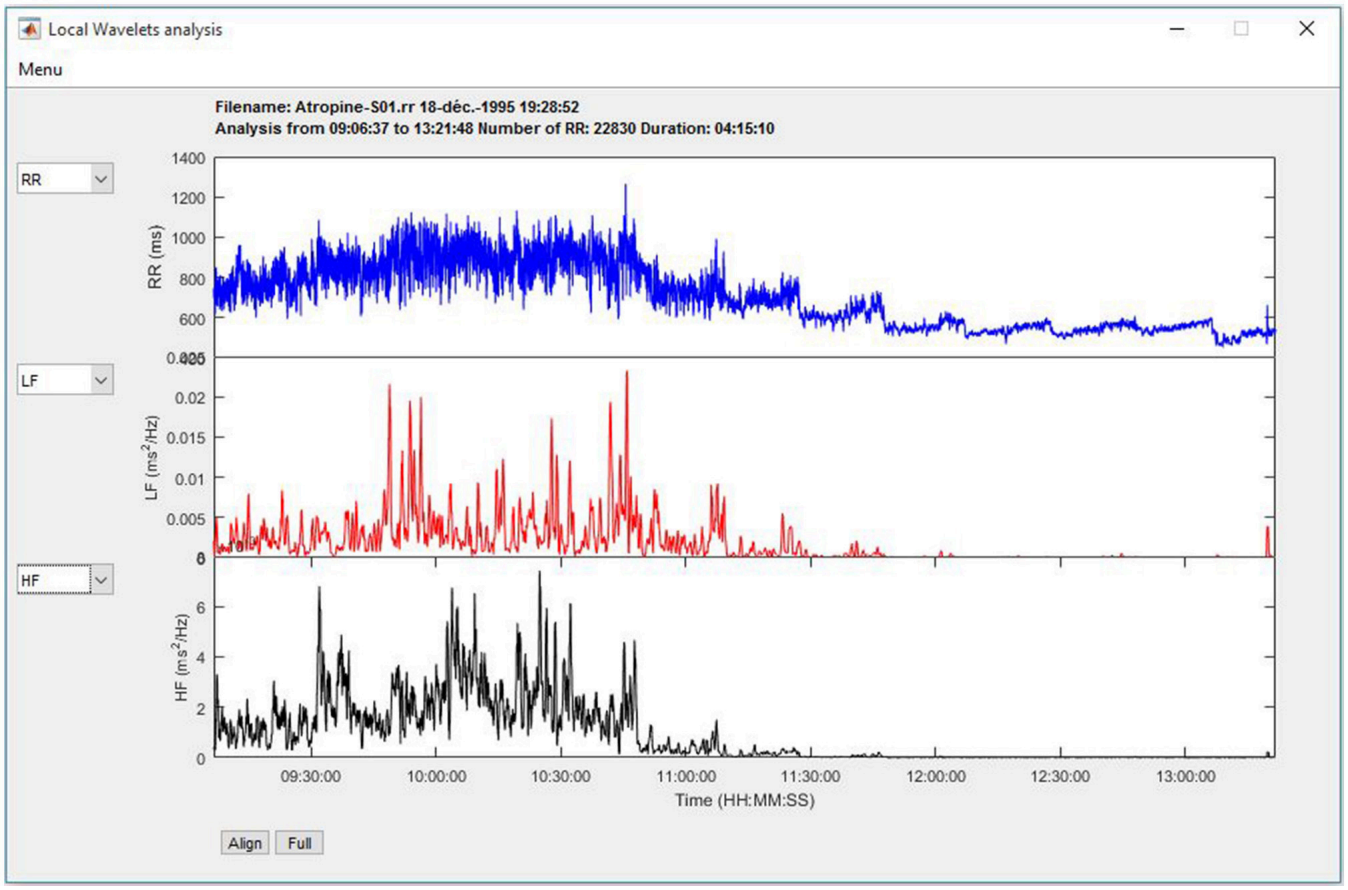
**Time-frequency analysis using Wavelet transform**.

The HRV indices are calculated using wavelet transform, as described in the Signal processing section. Three graphs allow plotting changes in RR, heart rate, LF, HF, LFnu, HFnu, and the LF/HF ratio along a time scale (Figure 7).

##### Analysis of surrounding events

**Figure 8:**
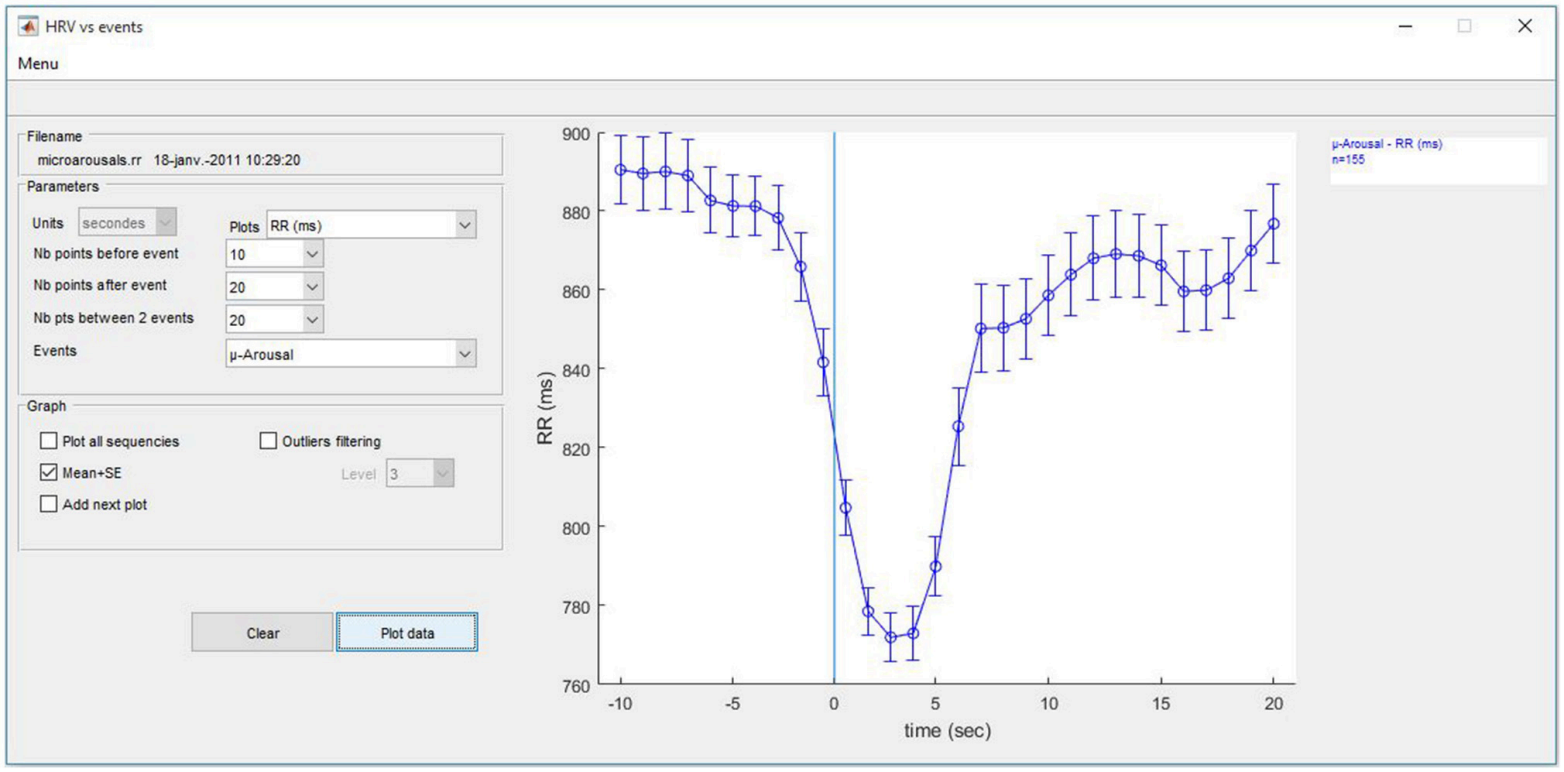
**Evolution of HRV around user-entered events**.

Another analysis is available for the cumulative changes in HRV indices surrounding a single event or repeatedly calibrated events entered by the user. The program searches all the sequences that meet the entered criteria and plots the results (Figure 8).

Possible indices are RR, heart rate, LF, HF, LFnu, HFnu, and the LF/HF ratio calculated using wavelet transform. All calculations are resampled at 1 Hz to allow plotting at 1-s steps. To allow comparisons, several options enable adding multiple analyses using different HRV indices and/or different events to the same graph. Results can be plotted with all sequences or on the mean ± SD, and simple outlier filtering can be applied.

#### Batch analysis

All the above-described HRV analyses can be performed in batch mode to enable processing a wide series of RR files without running repetitive operations. The results for all files found in the selected folder are then saved as.txt files, which are directly importable and ready for use in statistical software environments.

Five types of analysis are provided: Linear and nonlinear HRV (24 h/day/night), sequential HRV, HRV analysis of surrounding events, as described above, and, HRV analysis on all areas preset by the user.

## Hardware specifications and system requirements

*HRVanalysis* was developed using MATLAB 2016a and compiled using MATLAB compiler 6.2. MATLAB functions were used to develop the program, except for the calculation of entropy indices, Katz and Higuchi fractal indices, which were adapted from copyright files of Alvarez JM, the detrended fluctuation analysis, which was adapted from copyright files of Wenye G, the largest Lyapunov exponent which originates from copyright file of Wolf A, the Lampel-Ziv complexity index which comes from copyright files of Thai Q, and, the empirical mode decomposition which were adapted from copyright files of Rilling G and Flandrin P. It is not necessary to have MATLAB installed on the computer, because MATLAB Runtime v9.0.1 is packaged with the software, and is automatically installed if required. *HRVanalysis* works with Windows 64-bit operating systems.

To enable rapid plotting and calculation, it is recommended to have 2 GB of RAM and a minimal screen size of 1280 × 768.

## Sample runs

Some sample runs are distributed with the software package. These files are included to help the user get acquainted with the general functioning of the software and the included features.

## Availability, licensing and installation procedure

*HRVanalysis* is available free of charge for non-commercial use only (Freemium license, November the 9th 2015, under the number IDDN.FR.001.470001.000.S.P.2015.000.30000). Interested persons can download the software from the Web page at: https://anslabtools.univ-st-etienne.fr. Registration is requested so that users may be kept informed of free software updates.

The software is installed by running the Installer program and following the installation procedure. The program is delivered with a tutorial and sample runs. Bug reports, comments, and suggestions concerning the program can be emailed to ANSLabTools@univ-st-etienne.fr.

When using *HRVanalysis* to analyze data meant for publication, please cite this article and the software download webpage in the methods section. Please also credit the authors of the software when referencing it for the evaluation of the usefulness of the software and to add the reference to the *HRVanalysis* web pages.

## Conclusion and future directions

*HRVanalysis* was designed to meet laboratory requirements. It has been used and improved for over 20 years by the SNA-EPIS laboratory, Saint-Etienne, France. It has enabled the analysis and publication of HRV analyses for numerous purposes, including training and overtraining, cardiac and respiratory rehabilitation, sleep-disordered breathing, large cohort follow-ups, and children's autonomic status, pain (see references at https://anslabtools.univ-st-etienne.fr).

The main strength of *HRVanalysis* is its wide application scope. In addition to standard analysis, the software allows time-frequency analysis using wavelet transform as well as analysis of autonomic nervous system status surrounding scored events and on preselected labeled areas. Moreover, *HRVanalysis* is suitable for a considerable range of recording modes, from single recordings to large cohorts, and including batch signal processing.

*HRVanalysis* is meticulously maintained and developed for in-house laboratory use, and in response to users' comments and needs. Upcoming features to be developed in future versions include (1) more types of preformatted files for importing RR and EKG data; (2) selection of automatic RR correction levels, with specific thresholds for particular populations, such as newborns, babies, or athletes; (3) options to import events scored with other software such as polysomnography or polygraphy software, or from user-entered.txt files; (4) more time-frequency task options, such as selecting the wavelet shape and analysis level; and, (5) the addition of other significant cardiovascular signals such as blood pressure and respiration.

## Author contributions

Programmed of the software: VP. Design of the software: VP, FC, FR, JB. Debugged the software: VP, FC, SC, FR, JB. Wrote the paper: VP, FC. Managed the license agreement: FC. Managed the website: FC, VP. Revising the draft: FR, JB.

### Conflict of interest statement

The authors declare that the research was conducted in the absence of any commercial or financial relationships that could be construed as a potential conflict of interest.
